# Glutamate, aspartate and nucleotide transporters in the SLC17 family form four main phylogenetic clusters: evolution and tissue expression

**DOI:** 10.1186/1471-2164-11-17

**Published:** 2010-01-08

**Authors:** Smitha Sreedharan, Jafar HA Shaik, Pawel K Olszewski, Allen S Levine, Helgi B Schiöth, Robert Fredriksson

**Affiliations:** 1 Department of Neuroscience, Functional Pharmacology, Uppsala University, BMC, Uppsala SE 75124, Sweden; 2 Minnesota Obesity Center, Department of Food Science and Nutrition, Saint Paul, MN 55108, USA; 3 Department of Food Science and Nutrition, Saint Paul, MN 55108, USA

## Abstract

**Background:**

The SLC17 family of transporters transports the amino acids: glutamate and aspartate, and, as shown recently, also nucleotides. Vesicular glutamate transporters are found in distinct species, such as *C. elegans*, but the evolutionary origin of most of the genes in this family has been obscure.

**Results:**

Our phylogenetic analysis shows that the SLC17 family consists of four main phylogenetic clades which were all present before the divergence of the insect lineage. One of these clades has not been previously described and it is not found in vertebrates. The clade containing Slc17a9 had the most restricted evolutionary history with only one member in most species. We detected expression of Slc17a1-17a4 only in the peripheral tissues but not in the CNS, while Slc17a5- Slc17a9 are highly expressed in both the CNS and periphery.

**Conclusions:**

The *in situ *hybridization studies on vesicular nucleotide transporter revealed high expression throughout the cerebral cortex, certain areas in the hippocampus and in specific nuclei of the hypothalamus and thalamus. Some of the regions with high expression, such as the medial habenula and the dentate gyrus of the hippocampus, are important sites for purinergic neurotransmission. Noteworthy, other areas relying on purine-mediated signaling, such as the molecular layer of the dentate gyrus and the periaqueductal gray, lack or have a very low expression of Slc17a9, suggesting that there could be another nucleotide transporter in these regions.

## Background

Membrane proteins constitute about one third of all proteins encoded in the human genome [1]. The largest family of membrane-bound proteins consists of over 800 G protein-coupled receptors [2,3] while the second largest is the solute carrier (SLC) family including 384 human genes [4]. The SLC genes encode proteins related to passive transporters, ion-coupled transporters and exchangers. SLCs were functionally grouped into forty-three subfamilies [5]. Since then additional families have been added including five new subfamilies, SLC44 - SLC48, according to the Hugo Gene Nomenclature Committee [4].

A systematic phylogenetic analysis of the entire repertoire of SLC genes reveals that 15 of the SLC subfamilies, along with synaptic vesicle 2 (SV2) proteins, can be clustered into 5 major groups, named α-, β-, γ- and δ-groups [4]. The α-group is largest with SV2 proteins and seven SLC subfamilies (SLC2, 16, 17, 18, 22, 37 and 46). The main common features of the members of the α-group are the presence of 12 putative transmembrane (TM) regions, N- and C-termini at the cytosolic side, a large extracellular loop between TM1 and TM2 (except SLC17) and a large third intracellular loop.

The SLC17 family belonging to the α-group is known as the type I phosphate/vesicular glutamate transporter family [6]. The SLC17 family consists of nine genes that have previously been functionally divided into four subgroups: (i) type I phosphate transporters, SLC17A1-4, (ii) vesicular excitatory amino acid transporter, SLC17A5 (previously known as sialin) (iii) vesicular glutamate transporters (VGLUT), SLC17A6-17A8, and (iv) vesicular nucleotide transporter (VNUT), SLC17A9 [7,8].

The type I phosphate transporters are known to cotransport sodium (Na) and phosphate (Pi), with a capacity to also transport organic anions. Their ionic coupling properties have not been determined and the identity of their endogenous substrates remains unresolved. Moreover, data on the tissue distribution of the type I transporters seem rather limited. The SLC17A1 expression has been studied by northern blot and it has been identified in the kidney, liver and, at very low levels, in the brain [9,10]. The SLC17A2 has a different expression pattern, with relatively high levels in the heart and skeletal muscle and lower levels in the kidney, liver, lung, placenta, pancreas and brain [11,12]. SLC17A3 is limited to the liver and kidney [13], whereas SLC17A4 is expressed in the intestine, colon, liver, and pancreas [14].

The second group of SLC17 proteins consists of the vesicular excitatory amino acid transporter (VEAT/SLC17A5), previously known as sialin. It was first identified as a lysosomal sialic acid transporter implicated in the Salla disease and infantile sialic acid storage disorder [15]. However, a recent study showed that SLC17A5 serves as a vesicular protein transporting aspartate and glutamate and, hence, the name vesicular excitatory amino acid transporter (VAT) was suggested [7]. Slc17a5 shows ubiquitous expression [15]; in the brain it is predominantly expressed in the hippocampus, striatum and cerebral cortex [16,17].

The third group contains vesicular glutamate transporters involved in loading glutamate into synaptic vesicles of glutamatergic cells. In the SLC nomenclature the three identified VGLUT transporters, VGLUT1 through 3 are classified as Sl17A7, A6 and A8, respectively. Slc17a7 and Slc17a6 are predominantly expressed in glutamatergic neurons of the central nervous system (CNS). Interestingly, these genes have a complementary expression pattern with limited overlaps [18,19]. Unlike Slc17a7 and Slc17a6, Slc17A8 is expressed in CNS neurons not classically considered as glutamatergic [20,21]. The peripheral expression of Slc17a6 and Slc17a7 is limited to the pancreatic islets whereas Slc17a8 is expressed in the liver and, at lower levels, in the kidney.

The fourth type of the SLC17 genes is represented by a single gene in humans and it is named SLC17A9. Recently it has been identified as the first vesicular nucleotide transporter. The vesicular storage of this protein is confirmed by studies on PC12 cells. Functional characterization of SLC17A9 in liposomes containing the purified protein revealed that it transports nucleotides, such as ATP and ADP [8]. This is likely a major breakthrough in understanding neuronal signaling because ATP co-transmission is crucial in many neurons. The purinergic receptors that respond to ATP, UTP and adenosine serve as important drug targets and this transporter is likely to draw large attention for pharmaceutical development. Northern blot analysis showed wide expression of SLC17A9 in various organs, but predominantly in the brain, adrenals and the thyroid gland [8]. However, the detailed expression profile of SLC17A9 in the brain is unknown.

Vesicular glutamate transporters are fundamental components in glutamate signaling and are evolutionarily old. A vesicular glutamate transporter has been characterized in the tunicate *Ciona intestinalis *[22] as well as in *Drosophila melanogaster *[23] and *Caenorhabditis elegans *[24]. Also the presence of SLC17A9 in many animal species has been suggested [8]. However, despite the obvious biological importance of the SLC17 family, the evolutionary history of these genes has not been systematically mapped.

In the current project, we thoroughly mined the entire SLC17 family and explained the evolutionary events that shaped different branches of this family. We also performed comprehensive expression analysis of the nine SLC17 genes using quantitative real-time PCR (RT-PCR) in the rat. We then looked in more detail at newly discovered Slc17a9, the only known vesicular nucleotide transporter, and performed a comprehensive expression profiling of the Slc17a9 in the mouse brain by *in situ *hybridization.

## Methods

### Animal handling and tissue isolation for quantitative real-time PCR

Adult male Sprague-Dawley rats were housed in the controlled environment with optimum ventilation, temperature of 21°C, 12:12 LD cycle, and free access to standard chow and water. After the acclimation period of 7 days, the rats were sacrificed by decapitation. The peripheral tissues and brain regions of interest were isolated. These tissues were then immersed in the RNA-later solution (Ambion, USA) for 1 h at room temperature to remove RNases, and then stored at -80°C until further processed. All animal procedures were approved by the Uppsala Ethics Committee and followed the guidelines of European Communities Council Directive (86/609/EEC).

### RNA isolation and cDNA synthesis

RNA isolation and cDNA synthesis were performed as described previously [25]. In brief, the tissue was homogenized by sonication and the RNA was extracted using the TRIzol/chloroform method as previously reported [26]. Genomic DNA contamination was removed by adding DNaseI (Fermentas, Sweden). The cDNA was synthesized by priming with random hexameres and MMLV reverse transcriptase (GE Healthcare, Sweden)

### RT-PCR

The expression profiles of all the nine rat genes (rSlc17a1- rSlc17a9) were tested in a rat tissue panel containing cDNA of 16 different brain regions and various peripheral tissues. All the primers were designed using Beacon Primer Design 4.0 software (Premier Biosoft, USA) and all the sequences of primers used are shown in Additional File 1. The RT-PCR reaction mixture contained cDNA synthesized from 25 ng of total RNA, 0.25 pmol/μl of each primer, 20 mM Tris-HCl (pH 8.4), 50 mM KCl, 4 mM MgCl2, 0.2 mM dNTP, SYBR Green (1:50,000) (Invitrogen, USA) and 0.02 U/μl Taq DNA polymerase (Biotools, Spain). The reaction conditions were as follows: initial denaturation at 95°C for 4 min, followed by 50 cycles at 95°C for 15 s, 55-62°C for 30 s (optimal annealing temperature) and 72°C for 30 s. This was followed by a melt curve between 55°C and 97°C (10 seconds per step and 0.5°C increase per step) to identify non-specific amplification. All RT-PCR experiments were performed in duplicates. A negative control for each primer pair and a positive control with 25 ng of rat and mouse genomic DNA, respectively, was included on each plate. MyiQ thermal cycler (Bio-Rad Laboratories, Sweden) was used to perform RT-PCR.

### Data analysis and expression calculation

RT-PCR data were processed with the MyiQ software v1.04 (Bio-Rad Laboratories, Sweden). The melting curves were analyzed to confirm that only one product with the expected melting point had been amplified. The threshold cycle (Ct) values for the transcripts of interest were obtained and all samples with a signal, required to be at least 2 Ct values from the Ct value in the negative control (if any), were considered as expressed. LinRegPCR was used to calculate PCR efficiencies for each sample and Grubbs' test (GraphPad, USA) was applied to exclude the outliers and to calculate the average PCR efficiency for each primer pair. The GeNorm [27] protocol was used to identify the most stable housekeeping genes for each tissue and normalized quantities were calculated.

### cDNA Probe Synthesis

Antisense probe for mouse Slc17a9 was generated from EST clone 4986674 (Invitrogen). Plasmid preparation was performed using JETSTAR Plasmid Midi Kit (Genomed, USA). The plasmid were linearized with EcoRV (Fermentas, Sweden) and used as template for antisense digoxigenin (DIG)-labeled probe synthesis and T7 polymerase was used for probe synthesis. The sense probe was generated from the same EST clone and the plasmid was linearised with BstXI. Sp6 polymerase was used for digoxigenin (DIG)-labeled probe synthesis. The probe synthesis was carried out as described earlier [28].

### Tissue preparation for *in situ *hybridization

Adult male C57Bl6/J mice were housed in similar conditions as described above for the rats. The mice were anesthetized by an intraperitoneal injection of 1:1 mixture of Domitor (Medetomidine hydrochloride, 70 μg/g body weight, Orion, Finland) and Ketalar (Ketamine hydrochloride, 7 μg/g body weight, Pfizer, Sweden). Transcardial perfusion through the left ventricle with phosphate buffered saline (PBS) followed by freshly prepared 4% paraformaldehyde (PFA) was performed to obtain fixed brain tissue. The brain was excised and incubated in 4% formaldehyde overnight, thereafter the tissue was dehydrated and infiltrated with paraffin (Tissue Tek vacuum infiltration processor; Miles Scientific, Elkhart, IN). Paraffin-embedded brain was sectioned (7-μm thick) on the Microm microtome onto Superfrost slides (Menzel-Gläser, Braunschweig, Germany) and stored at 4°C until usage.

### *In situ *hybridization

The paraffin sections were deparaffinized in X-tra solve (Medite Histotechnic, Burgdorf, Germany) and rehydrated with the series of ethanol washes (100%, 90%, 70%, 50%, 25%); followed by washing in PBS. The rehydrated sections were fixed with 4% PFA for 10 min, washed with PBS and treated with proteinase K (Invitrogen, Germany); 27 μg/ml diluted in 10 mM Tris-HCl/1 mM EDTA, pH 8.0) for 15 min. The sections were refixed with 4% PFA for 5 min and acetylated for 10 min in 1.3% triethanolamine (Sigma-Aldrich, USA) 0.2% acetic anhydride (Fluka, Neu-Ulm, Germany), and 0.06% HCl diluted in RNase free water. The sections were then permeabilized with 1% Triton-X 100 (Sigma-Aldrich, USA) for 30 min and washed with PBS. The slides were subsequently incubated in the hybridization buffer [50% formamide (Fluka, Germany), 5× SSC, 5× Denhardt's, 250 g/ml yeast transfer RNA (Sigma-Aldrich, USA), 500 g/ml sheared salmon sperm DNA (Ambion, Austin, TX) diluted in RNase free water] without the probe for 1-2 h. The probe (0.5 μg/200 μl) was denatured in the hybridization buffer at 80°C for 5 min and then cooled in ice. The prehybridized sections were hybridized with the cooled denatured probe in the hybridization buffer overnight at 65°C. Next day the slides were transferred to a 0.2× SSC and incubated at 65°C for 1-3 hrs. The slides were then washed first with 0.2× SSC at room temperature and then in the B1 solution (0.1 M Tris-HCl, pH 7.5, and 0.15 M NaCl). The sections were immunoblocked with 10% fetal calf serum in B1 and incubated overnight at 4°C in peroxidase conjugated anti-digoxigenin Fab fragments (Roche, Mannheim, Germany) diluted 1:500 in B1 containing 10% fetal calf serum. The following day the slides were washed with PBS and then incubated in 300 μl of the amplification buffer (Molecular probes™ Tyramide Signal Amplification Kit, Invitrogen, USA) containing 3 μl of the labeled tyramide solution (Molecular probes™ Tyramide Signal Amplification Kit, Invitrogen, USA ) for 10-15 min. The sections were washed with PBS and stained with DAPI. They were mounted using DTG (2.5% DABCO (Sigma), 50 mM Tris-HCl pH 8.0, 90% glycerol) and analyzed using the fluorescent microscope (Zeiss XBO75). The Slc17a9 probe covers the 1178 bp of the entire cDNA.

### Data mining

The sequences for the 9 human SLC17 family proteins were downloaded from the NCBI http://www.ncbi.nlm.nih.gov/ using the Entrez data retrieval tool with the accession numbers as follows: SLC17A1, NP_005065.2; SLC17A2, NP_005826.1; SLC17A3, NP_001091956.1; SLC17A4, NP_005486.1; SLC17A5, NP_036566.1; SLC17A6, NP_065079.1; SLC17A7, NP_064705.1; SLC17A8, NP_647480.1; SLC17A9, NP_071365.3. The sequences from each family were aligned using Tcoffee 5.72 [29]. From the alignments, sequence Hidden Markov Models (HMMs) were constructed using the HMMER 2.2 package [30]. The models were constructed using HMMbuild with default settings and calibrated using HMMcalibrate. The models were searched against the following protein datasets: *Mus musculus *(NCBI build 37.50, pep all); *Gallus gallus *(Hashington University build 2.50, pep all); *Tetraodon nigviridis *(build 8.5, pep all); *Danio rerio *(build 7.50, pep all); *Takifugu rubripes *(build 4.50, pep all); *Ciona savigny *(build 2.0.50, pep all); *Branchiostoma floridae; Drosophila melanogaster *(4.50, pep all); *Caenorhabditis elegans *(build 190.50, pep all); *Schizosaccharomyces pombe *(pompep); *Saccharomyces cervisiae *(build 1.01.50, pep all). All hits with an e-value better than 0.1 were extracted and non-SLC17 proteins were removed using phylogenetic analysis. The analysis included all human SLC16, SLC17, SLC18 and SLC22 proteins in order to represent the SLC families most similar to SLC17 [4].

### Phylogenetic analysis

Phylogenetic trees were calculated for the SLC17 family using the following procedure. Amino acid sequences in Fasta format were aligned using T-Coffee 5.72. The alignment was bootstrapped 1000 times using SEQBOOT from the Win32 version of the Phylip 3.6 package [31]. Maximum-parsimony trees were calculated on the bootstrapped alignment with PROTPARS from the Win32 version of the Phylip 3.6 package. The trees were un-rooted and calculated using ordinary parsimony, and the topologies were obtained using the built-in tree search procedure. Majority-rule consensus trees were constructed using CONSENSE from the Win32 version of the Phylip 3.5 package. Neighbor joining trees were calculated on the same bootstrapped sequences using the Phylip 3.6 programs PROTDIST followed by NEIGHBOR. For the maximum likelihood tree, the topology obtained from the maximum parsimony tree was used as a user-defined tree in TreePuzzle [32] and maximum likelihood branch lengths were estimated in TreePuzzle using the following parameters: Type of analysis: Tree reconstruction; Tree search procedure: User defined trees; Compute clocklike branch lengths: No; Location of root: Best Place (automatic search); Parameter estimates: Exact (slow); Parameter estimation uses: 1st input tree; Type of sequence input data: Amino acids; Model of substitution: JTT. Amino acid frequencies: Estimate from data set; Model of rate heterogeneity: Mixed (1 invariable + 8 Gamma rates); Fraction of invariable sites: Estimate from data set; Gamma distribution parameter alpha: Estimate from data set; Number of Gamma rate categories: 8. The trees were plotted using TreeView [33].

## Results

### Mining

**Figure 1:**
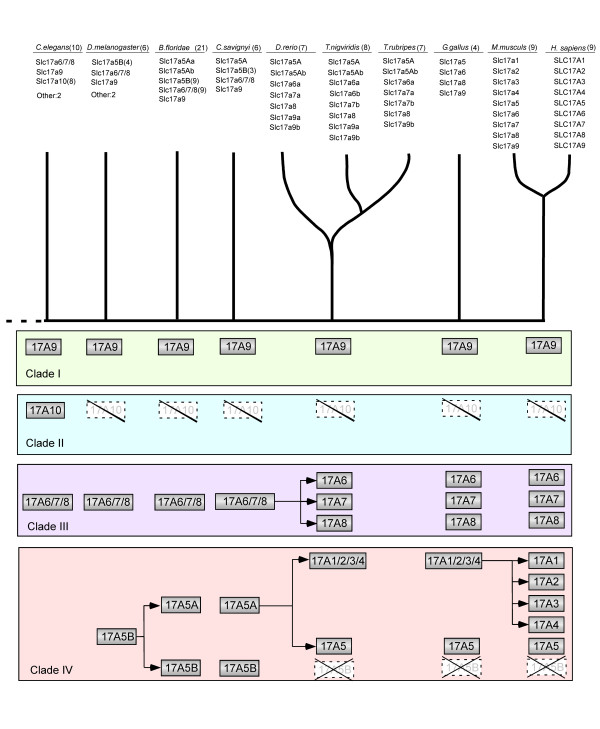
**The evolutionary history of the SLC17 family as deduced from the phylogenetic analysis**. The top table describes which genes (their number in the parenthesis) were identified in each species. The middle part indicates the interrelationship of the species investigated in this study while the bottom panel indicates which genes were most likely present before the split of the lineage leading to the respective species.

**Figure 2:**
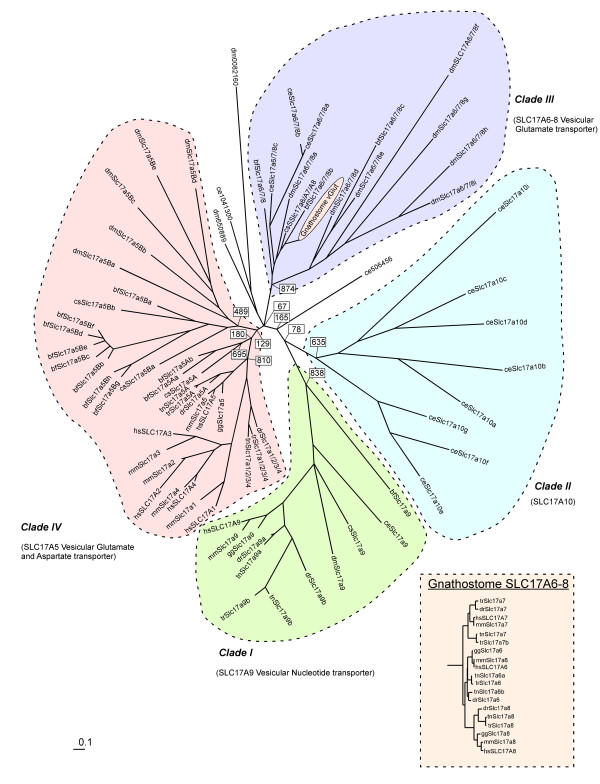
**The phylogenetic tree of the SLC17 family from human (hs, *Homo sapiens*), chicken (gg, *Gallus gallus*), puffer fish (tn, *Tetraodon nigroviridis*), fugu fish (tr, *Takifugu rubripes*), zebra fish (dr, *Danio rerio*), amphioxus (bf, *Branchiostoma floridae*), sea squirt (cs, *Ciona savigyni*), fruit fly (dm, *Drosophila melanogaster*) and round worm (ce, *Caenorhabditis elegans*)**: Our phylogenetic analysis shows that the SLC17 family consists of four main clades termed III, III and IV with clade I containing two ancient sub-clades. The topology for the tree is calculated from a maximum parsimony (MP) tree and the branch lengths are estimated on that topology using the maximum likelihood method and are proportional to the evolutionary distance. Numbers in boxes indicates MP bootstrap values for the nodes close to the root of the tree. The vGlut family from gnathostomes is removed from the main tree and presented as an insert for clarity although the phylogeny was calculated for the tree including the gnathostome vGluts. Branch lengths for the two trees are drawn using the same scaling.

We mined the entire datasets of predicted proteins from eight animal species (see Figure 1) as well as two unicellular species of yeast for SLC17 proteins using a sequence Hidden Markov Model (HMM) trained on the alignment containing the nine human SLC17 protein sequences. We combined the hits with all human SLC16, SLC17, SLC18 and SLC22 proteins and used phylogenetic analysis to remove all non-SLC17 proteins. The coding regions from all predicted proteins were manually inspected and curated, essentially using the same approach as previously described [34]. The SLC17 proteins identified in the mining were combined with all human SLC17 proteins into the phylogenetic analysis. This phylogenetic analysis shows that the SLC17 family forms four main clades (see Figure 1). One of these is only found in the invertebrate *C. elegans *and we suggest that this family be called Slc17a10 (clade II) to adhere to the previous nomenclature, see Additional File 2 for a primary sequence comparison with the human SLC17 sequences. The Slc17a9, known as a vesicular nucleotide transporter [8], forms a separate clade (clade I) and places most closely to the Slc17a10 clade. This clade I has one member in each animal species, with the exception of the three species of teleost fish investigated, which appear to have one extra copy, most likely originating from the whole genome duplication that occurred before the divergence of the teleost fish [35]. The *B. floridae *Slc17a9 places most basal in the clade I, outside *D. melanogaster *and *C. elegans*. This is surprising considering the species relationship, but the Slc17a9 appears to have acquired lineage-specific changes in the *B. floridae*, which reflects its basal position in the tree. Clade III contains the human SLC17A6, A7 and A8, the vesicular glutamate transporters. The phylogenetic analysis shows that the three vesicular glutamate transporters found in humans originate from a common ancestor before the split of gnathostomes (Figure 2). It is also apparent that extra copies of Slc17a6 and Slc17a7, which probably were formed in the whole genome duplication before the divergence of teleosts, were retained in some teleost species. Also Slc17a7 appears to have been lost in the chicken lineage. There is most likely one ancestral gene to the gnathostome SLC17A6 - A8, as we found protein sequences placing basal of this group in *B. floridae*, *D. melanogaster*, *C. savygni *and *C. elegans*. This sequence seems to have duplicated locally in *C. elegans *to form three copies (denoted a - c). Moreover, there is a basal group containing sequences from *B. floridae *and *D. melanogaster*, which also seems to have expanded locally in both species, which could be ancestral. If this is the case, these genes must have been lost after the divergence of *B. fluviatilis*, but it is also possible that these sequences are copies of the same ancestral sequence as the gnathostome Slc17a6 - a8, but form their own group due to rapid evolution. The largest clade contains five human SLC17 sequences (clade IV), SLC17A1 - A5, where SLC17A5 is clearly the most basal member. This clade IV consists of two subgroups, which we denote SLC17A5A and SLC17A5B, where the human SLC17A5, the vesicular excitatory amino acid transporter [7] belongs to the B group, whereas the A group appears to have been lost before the appearance of vertebrates. In this clade, Slc17a1 - a4 appear to be the most recent members and we see two possible scenarios for their evolution (see Discussion). These two scenarios suggests that Slc17a1 - a4 are either specific to mammals, originating from Slc17a5, or that they arose from a common ancestor present before the divergence of bony fish, that was lost in the bird lineage. Regardless of the evolutionary history, these four orphan transporters are likely to serve a specific function or, at least a more developed one, in mammals. In this clade IV, we also see two large expansions, one in the *B. fluviatilis *which includes eight members of the A group and two members of the B group, and one in *D. melanogaster *which has five members of the A group. We found no members of the Slc17a5 clade in *C. elegans*.

### RT-PCR measurements

Expression analysis of all nine members of the SLC17 family was performed on a panel of rat tissues. The rat panel consisted of cDNA synthesized from the RNA isolated from seven coronal sections of the rat brain [25], different regions of the brain, various peripheral tissues in total of 35 samples. The normalization factor was calculated based on the expression of four housekeeping genes and the relative expression values are the fold increase from the minimum detected expression in the rat panel. The validation of the method and use of normalization factors are explained elsewhere [36].

The Slc17a1 and Slc17a2 were expressed in the skeletal muscle, kidney and liver. Slc17a3 was expressed in the skeletal muscle, kidney and uterus, whereas Slc17a4 was expressed in the kidney, liver, stomach and intestine. Slc17a5 had ubiquitous expression in all tissues tested. Slc17a6 and Slc17a7 showed expression at different yet detectable levels in all brain regions tested and in the spinal cord. However, in the periphery Slc17a6 was expressed in the eye, skeletal muscle, liver, spleen and thymus, while SLC17A7 was detected in the eye and heart. Slc17a8 was predominantly expressed in the choroids plexus and - at low levels - in the cortex, hypothalamus, hippocampus, brainstem, spinal cord, stomach and eye. Slc17a9 expression appeared ubiquitous with the highest level in the stomach, intestine, liver, skeletal muscle, spleen and blood; relatively lower levels were detected in all brain regions studied, spinal cord, adrenal gland, eye, skin and skeletal muscle.

**Figure 3:**
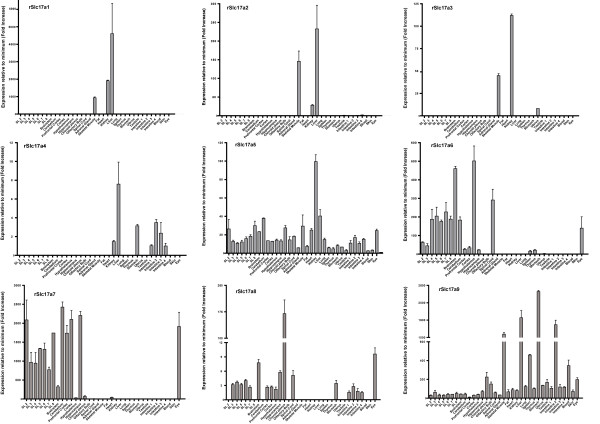
**Expression data of the SLC17 family**. Relative expression obtained by quatitative real-time PCR for the rat SLC17 family: rSLC17A1, rSLC17A2, rSLC17A3, rSLC17A4, rSLC17A5, rSLC17A6 rSLC17A7, rSLC17A8 and rSLC17A9. Error bar displays the standard deviation. The x-axis on all the graphs indicates the tissues tested. SL2-SL8 indicate the coronal section of the rat brain [25]. Note that the scales on y-axis are varied in the graphs.

The expression patterns of the nine genes (Slc17a1- Slc17a9) in the brain obtained from RT-PCR (Figure 3) were compared to the murine *in situ *hybridization data from the Allen Brain Atlas and were found to be in good agreement (see Additional file 2).

### *In situ *hybridization analysis for Slc17a9

**Figure 4:**
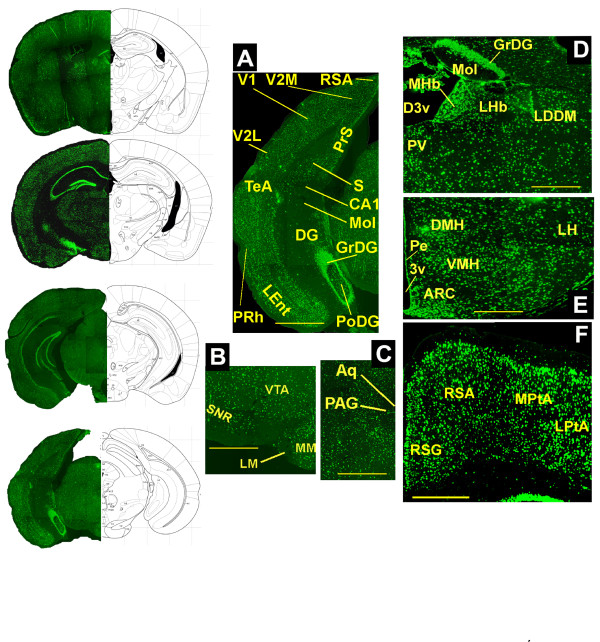
**Detection of SLC17A9 mRNA in the coronal sections of the mouse brain by fluorescent *insitu *hybridisation of Slc17a9 antisense probe**: A- F represents the magnified images of the cortex, hippocampus thalamus and the hypothalamus. Scale bar: A-1 mm, B & C-0.7 mm, D, E & F-0.5 mm. 3v - third ventricle, ARC - arcuate nucleus, Aq - aqueduct, CA1 - field CA1, D3v - third ventricle, dorsal part, DG - dentate gyrus, DMH - dorsomedial hypothalamic nucleus, GrDG, dentate gyrus, granular layer, LDDM - lateral dorsal nucleus of the thalamus, dorsomedial part, LEnt - entorhinal area, lateral part, LH - lateral hypothalamic area, LHb - lateral habenular nucleus, LM - lateral mammillary nucleus, LPtA - lateral parietal association cortex, MHb - medial habenular nucleus, MM - medial mammillary nucleus, Mol - molecular layer of the dentate gyrus, MPtA - medial parietal association cortex, PAG - periaqueductal gray, Pe - periventricular nucleus, PoDG - dentate gyrus, polymorph layer, PRh - perirhinal cortex, PrS - presubiculum, PV - paraventricular nucleus of the thalamus, RSA - rostrosplenial agranular cortex, RSG - retrosplenial granular cortex, S - subiculum, SNR - substantia nigra, TeA - temporal association cortex, V1 - primary visual cortex, V2M - secondary visual cortex, medial, VMH - ventromedial hypothalamic nucleus, VTA - ventral tegmental area. Scale bar: A-1 mm, B & C-0.7 mm, D, E & F-0.5 mm

*In situ *hybridization revealed that Slc17a9 mRNA is widely expressed in the brain (Figure 4). It is particularly abundant throughout the cerebral cortex, where the distribution is uniformly high. In the hippocampus, the granular layer of the dentate gyrus (DG) displays a remarkably high level of staining, while the molecular layer of the DG and CA1 are devoid of the Slc17a9 signal and polymorph cells in the DG show a relatively weak expression of this gene. Also, little staining was visible in the periaqueductal gray (PAG). In the hypothalamus, the arcuate nucleus (ARC) and the cells immediately adjacent to the third ventricle (in the periventricular area; Pe) appear particularly rich in Slc17a9 mRNA. In addition, a strong signal was detected in the ventromedial (VMH) and dorsomedial (DMH) nuclei. Finally, in the thalamus, very high levels of Slc17a9 mRNA are found in the medial habenular nucleus (MHb). A sense probe was generated for the Slc17a9 as a negative control and it inferred no signal.

## Discussion

We have performed detailed mining and phylogenetic analysis of all SLC17 family proteins from eight species. The analysis shows that this family consists of four main phylogenetic clades, which were present before the divergence of the insect lineage from those leading to mammals. We denote those clades I to IV. Three of these main clades include all the human members while one clade contains only sequences from *C. elegans*. No member of this entire clade has previously been described in the literature. We propose the order of events for the evolutionary history for the SLC17 family from the nematodes to human and this is schematically presented in Figure 1. We did not find any SLC17 family genes in any species more basal than the nematode, although we found sequences for predicted proteins in *Nematostella vectensis *that have similarity to the SLC17 family as well as to other SLC α-family members. Our current data suggest that the four main ancestral groups, denoted in four main colors in Figure 2, originated from an MFS type transporter [4] before the divergence of nematodes.

Clade I contains only the newly discovered vesicular nucleotide transporter, VNut [8]. The evolutionary history of this gene is unique within the SLC17 family as it is the only gene that has one member in almost all the species. For multiple sequence alignment of the VNuts discussed in this paper, see Additional File 3 and Additional file 4. An exception is however among bony fish, where *T. nigroviridis *and *D. rerio *have retained an extra copy, most likely as a result of the extra genome duplication. The new clade II was only found in *C. elegans*. Our searches and phylogenetic analysis suggest that these sequences are indeed members of a distinct clade that does not exist in the vertebrate linage. Clade III contains the vesicular glutamate transporters, VGluts, which were present already in *C. elegans*. A common ancestor to the three mammalian VGluts appears to have existed before the divergence of gnathosomes and this common ancestor likely gave rise to the three VGluts found in current vertebrates (Figure 2). The invertebrate species and also *B. floridae *have more than one gene in Clade III; the phylogenetic analysis (Figure 1) suggests that this could be the outcome of local duplications. Clade IV has the most complex evolutionary history of the four SLC17 clades. The first members of this clade appear in *D. melanogaster *having five local copies of the original Slc17a5 sequence, which we name Slc17a5B. This gene gave rise to another Slc17a5 subtype, Slc17a5A, before the split of the *B. floridae *lineage. The original copy, Slc17a5B, was lost before the split of bony fish. Also, before the split of bony fish, Slc17a5 was duplicated to form the ancestor of Slc17a1 - a4 that we named Slc17a1/2/3/4, which we found in bony fish.

The long evolutionary history of the VNut suggests that nucleotides as neurotransmitters are fundamental to neuronal signaling and that this transport mechanism is likely to have been present already in the most primitive nervous systems. In vertebrates, nucleotides are known to function as co-transmitters to classical transmitters, in particular GABA [25]. Purines have also been shown to be released as neurotransmitters on their own and from their own vesicles, but at the same synapses as other transmitters, inducing excitatory postsynaptic currents. This has been most clearly demonstrated in fish [37]. Nucleotides as functional neurotransmitters act on two classes of purinergic postsynaptic receptors, the G protein-coupled P2Y [38] and the ionotropic P2X receptors [39]. Among these, the P2Y receptors have the shortest evolutionary history, as being present in fish [40], but not in *B. floridae *[41]. The evolutionary history of the P2X receptors is, however, much longer. P2X receptors are found in algae [42] as well as in *Dictyostelium discoideum *[43]. Interestingly, these receptors appear to have been lost independently in both the arthropod and nematode lineages [44]. Here we show that although neither arthropods nor metazoans appear to have purinergic receptors, at least not the type present in vertebrates, these lineages have retained a single copy VNut transporter. These data suggest that purinergic signaling was present already in early eukaryotes before the divergence of the plant lineage and lineage leading to metazoans.

We observe that rat Slc17a9 in the present study shows a differential expression profile when compared to the mouse and the human counterpart in an earlier study [8]. This discrepancy can be due to the species specific expression pattern of the Slc17a9 gene which has also been observed earlier between mouse and human [8]. The RT-PCR data infers that the expression of the adrenal gland is 34-fold higher than the tissue that showed lowest expression. Further, the difference in the method of detection of the mRNA level could account for some changes in the expression levels.

Postsynaptic currents induced by stimulation of purinergic receptors have been demonstrated functionally in the locus coeruleus, hippocampus, hypothalamus, dorsal horn, habenuclear nucleus of the thalamus and somatosensory cortex in mammals [45]. It is however unclear whether all brain regions and most neurons use ATP as a cotransmitter. Our *in situ *hybridization shows that SLC17A9 mRNA is widely expressed in the mouse brain, suggesting that neurons from most brain regions may have the capacity to store ATP in vesicles. This widespread distribution is in concert with involvement in multiple processes suggested for purines, including those related to feeding regulation [46], stress and anxiety responsiveness [47,48], capacity of receptors to bind amino acids [49], and hippocampal neural network formation [50]. It also shows that regions where purinergic neurotransmission has previously been demonstrated, for example, medial habenula and the dentate gyrus of the hippocampus, have particularly high expression of Slc17a9 (see Figure 4). It is however interesting to note that some regions, such as the molecular layer of the dentate gyrus and the PAG, seem to lack or have very low expression of Slc17a9. Since purinergic neurotransmission has been reported within these areas [47,50,51], it is likely that SLC17A9 does not serve as the sole vesicular nucleotide transporter in these regions. The remaining orphan transporters in the SLC17 family are Slc17a2, a3, and a4 which are expressed only in the periphery (Figure 3) and hence it is unlikely that these would be the additional transporters for purinergic neurotransmission. One possibility is that one of the six orphan transporters phylogenetically closely related to the SLC16, SLC17 and SLC18 families, that we recently reported [4], could serve as an additional vesicular nucleotide transporter. Another possibility is that SLC17A9 accounts for the only *vesicular *nucleotide transporter and those neurons that do not express this gene do not use purinergic signaling.

The three orphan transporters Slc17a2 - 4 [6] and Slc17a1 were found only in mammals and originated from the common ancestor, Slc17a1/2/3/4, which we found in all three species of teleost fish. The duplications from this common ancestor occurred most likely after the split of the bird lineage as we did not find the common ancestor or any of the duplicates. The basal member of this clade IV; Slc17a5 was recently shown to be a vesicular transporter for glutamate and aspartate, hence it is possible that SLC17A1 to A4 could also be vesicular transporters. In fact, all transporters from the SLC17 family except SLC17A1 which transport p-aminohippuric acid across renal apical membrane[52] that have been functionally investigated are to our knowledge - a vesicular transporter, which suggests that the location to vesicles is an ancestral functional feature common for the SLC17 family. Our quantitative RT-PCR data show that Slc17a1, a2, a3 and a4 are not expressed in the brain, which was confirmed by searches in the Allen Brain Atlas database [53]. These transporters are mainly expressed in the muscle, liver and kidney, though it is still possible that these are expressed in secretory granules or other vesicles in these tissues. The SLC17A9 protein was, for example, shown to be expressed on chromaffin granules in the mouse adrenal gland [8].

## Conclusion

We have performed extensive mining of a cluster of solute carriers, named SLC17, that transport glutamate, aspartate and nucleotides. We show the presence of four independent evolutionary clades among these genes which correspond well to the currently known substrates. Surprisingly we found a new branch that forms one of these main clades including eight novel genes in *C. elegans*. The overall tissue distribution in central and peripheral panels provides for the first time a comprehensive overview of the expression patterns for this family. The detailed *in situ *hybridization of SLC17A9 shows high expression throughout the cerebral cortex and distinct patterns in, for example, the medial habenula and the dentate gyrus of the hippocampus where purinergic neurotransmission is important. Other regions implicated in purine signaling, such as the molecular layer of the dentate gyrus and the PAG, seem to lack or have very low expression of Slc17a9 suggesting that there could be another nucleotide transporter in these regions.

## Authors' contributions

SS, JHAS performed the in situ and tissue panel PCR. RF studied the evolutionary aspects of the SLC17 family. PKO, SS, HBS and RF analyzed the data. SS, ASL, RF and HBS prepared the manuscript and managed the study. All authors read and approved the final manuscript.

## Supplementary Material

Additional file 1 **Primer sequences**. PCR primers used for the quantitative realtime PCR assays. Click her for the file

Additional file 2 **Expression of the SLC17 family in mouse according to the Allen Brain Atlas resource**[53]. Expression levels of the SLC17 family sequences in mouse brain. Numbers between 0 (no expression) and 100 (highest expression) are derived from the database using the Expression Level feature. Click her for the file

Additional file 3 **Alignment of dm Slc17a10 and the all human SLC17 sequences**. Alignment of protein sequences from human (hs, *Homo sapiens*) hsSLC17A1-17A9 and fruit fly (dm, *Drosophila melanogaster*) dmSlc17a10. TM represents the predicted transmembrane regions. Click her for the file

Additional file 4 **Alignment of protein sequences of SLC17A9 from various species**. Alignment of protein sequences of SLC17A9 from human (hs, *Homo sapiens*), chicken (gg, *Gallus gallus*), puffer fish (tn, *Tetraodon nigroviridis*), fugu fish (tr, *Takifugu rubripes*), zebra fish (dr, *Danio rerio*), amphioxus (bf, *Branchiostoma floridae*), sea squirt (cs, *Ciona savigyni*), fruit fly (dm, *Drosophila melanogaster*) and round worm (ce, *Caenorhabditis elegans*). TM represents the predicted transmembrane regions. In the sea squirt (cs, *Ciona savigyni*), sequence X indicates the peptide sequence X:QGQLYLLYGVLDNELYNKFVICPQTFLFG. Click her for the file
