# Supplementary material for: Glutamate, aspartate and nucleotide transporters in the SLC17 family form four main phylogenetic clusters: evolution and tissue expression
Source: BMC Genomics. 2010 Jan 8;11:17. doi: 10.1186/1471-2164-11-17 (PMC2824716; doi:10.1186/1471-2164-11-17)
Supplement: Additional file 1 — Primer sequences. PCR primers used for the quantitative realtime PCR assays. [file 1471-2164-11-17-S1.doc]

|  | Gene | Forward primer | Reverse Primer | Annealing temperature (ºC) |
| --- | --- | --- | --- | --- |
| 1 | Slc17a1 | TGAGCAGCAGTGAGAAGG | GGCGTGTATGTAACCAGAAG | 60,1 |
| 2 | Slc17a2 | CACCAACTGCTGCCTGAG | AAATCACTTTCACCACGACAG | 60,1 |
| 3 | Slc17a3 | CAGGAATGATATTGGGAATCTTTG | CAGAGAAGGCAGCAGACC | 55,7 |
| 4 | Slc17a4 | CGTTCCTTGTTCTTATTGC | GATGTATCTCTTCTCACCAG | 55,7 |
| 5 | Slc17a5 | GGAAACGATGATGAGGAAAG | AGTGCTGTTGGAATCTACC | 55,7 |
| 6 | Slc17a6 | TGGTTGCGTTAAGACTTC | CCACGGGTTTCCTAATAG | 55,7 |
| 7 | Slc17a7 | CTGTTCTGGTTGCTTGTC | CAGGGTGTGTTAAACTTC | 55,7 |
| 8 | Slc17a8 | GGAAGGAAGGAGTGAAGAATGC | GCTCGGGCTCTGGATGTC | 55,7 |
| 9 | Slc17a9 | CCACCTCGGATGTATGAGCAGAC | CCCACTGAACCTGAGAGAAGCC | 61,3 |
