# Supplementary material for: Glutamate, aspartate and nucleotide transporters in the SLC17 family form four main phylogenetic clusters: evolution and tissue expression
Source: BMC Genomics. 2010 Jan 8;11:17. doi: 10.1186/1471-2164-11-17 (PMC2824716; doi:10.1186/1471-2164-11-17)
Supplement: Additional file 2 — Expression of the SLC17 family in mouse according to the Allen Brain Atlas resource[53]. Expression levels of the SLC17 family sequences in mouse brain. Numbers between 0 (no expression) and 100 (highest expression) are derived from the database using the Expression Level feature. [file 1471-2164-11-17-S2.doc]

|  | Slc17a1 | Slc17a2 | Slc17a3 | Slc17a4 | Slc17a5 | Slc17a6 | Slc17a7 | Slc17a8 | Slc17a9 |
| --- | --- | --- | --- | --- | --- | --- | --- | --- | --- |
| Olfactory bulb | No Data | 0 | 3 | 0 | 20 | 25 | 100 | 4 | No Data |
| Cerebral cortex | No Data | 0 | 2 | 0 | 15 | 8 | 100 | 5 | No Data |
| Hippocampus | No Data | 0 | 2 | 0 | 13 | 13 | 100 | 5 | No Data |
| Striatum | No Data | 0 | 0 | 0 | 3 | 10 | 21 | 12 | No Data |
| Thalamus | No Data | 0 | 0 | 0 | 3,4 | 100 | 100 | 6 | No Data |
| Hypothalamus | No Data | 0 | 0 | 0 | 1,1 | 80 | 8 | 4 | No Data |
| Midbrain | No Data | 0 | 1 | 0 | 1,3 | 100 | 15 | 7 | No Data |
| Medulla | No Data | 0 | 3 | 0 | 6,0 | 100 | 53 | 4 | No Data |
| Pallidum | No Data | 0 | 7 | 0 | 1,8 | 18 | 12 | 13 | No Data |
| Cerebellum | No Data | 0 | 6 | 0 | 4 | 21 | 100 | 3 | No Data |
| Pons | No Data | 0 | 0 | 0 | 3,0 | 100 | 53 | 11 | No Data |
