# Supplementary figures and images for: Glutamate, aspartate and nucleotide transporters in the SLC17 family form four main phylogenetic clusters: evolution and tissue expression
Source: BMC Genomics. 2010 Jan 8;11:17. doi: 10.1186/1471-2164-11-17 (PMC2824716; doi:10.1186/1471-2164-11-17)

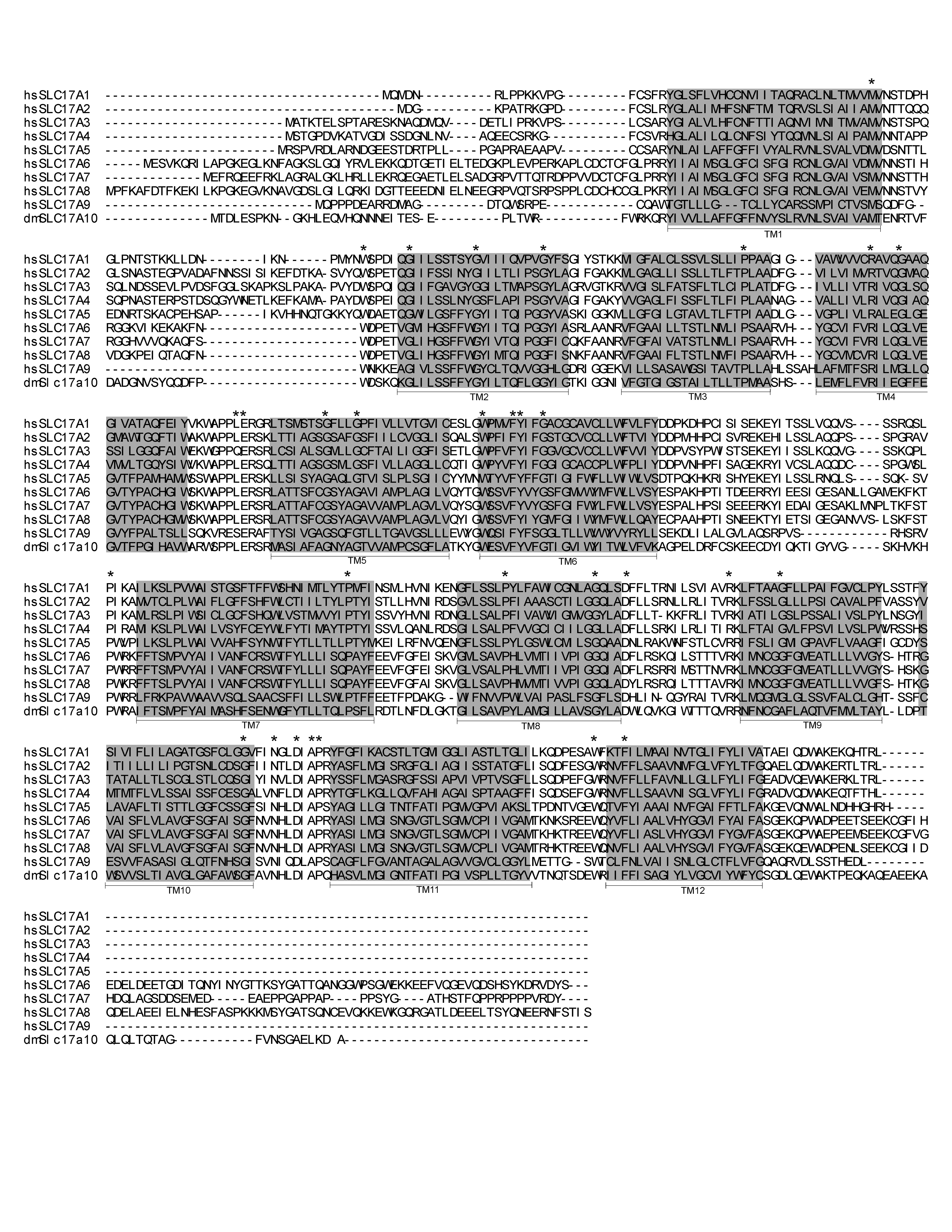

Supplement: Additional file 3 — Alignment of dm Slc17a10 and the all human SLC17 sequences. Alignment of protein sequences from human (hs, Homo sapiens) hsSLC17A1-17A9 and fruit fly (dm, Drosophila melanogaster) dmSlc17a10. TM represents the predicted transmembrane regions. [file 1471-2164-11-17-S3.tiff]

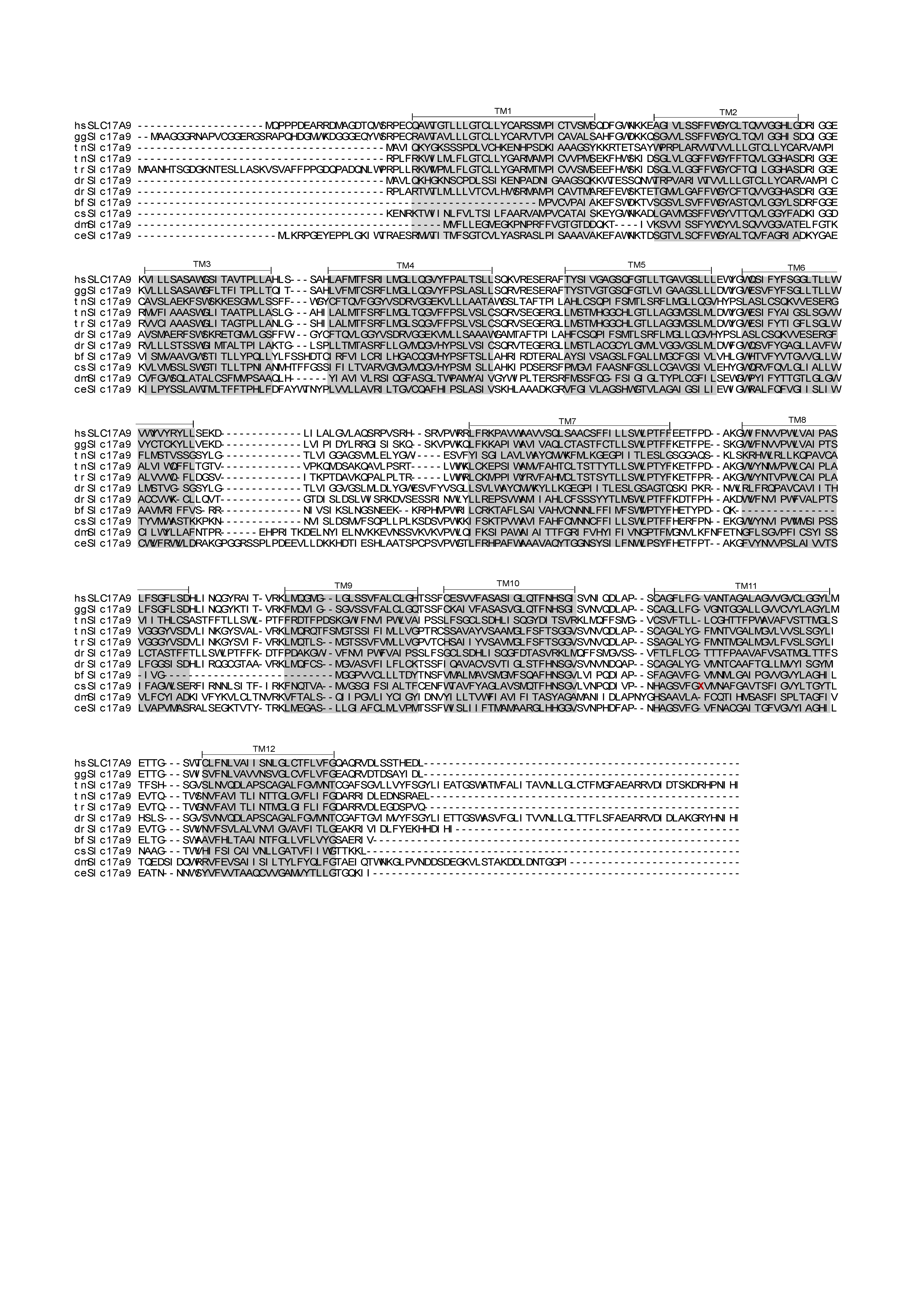

Supplement: Additional file 4 — Alignment of protein sequences of SLC17A9 from various species. Alignment of protein sequences of SLC17A9 from human (hs, Homo sapiens), chicken (gg, Gallus gallus), puffer fish (tn, Tetraodon nigroviridis), fugu fish (tr, Takifugu rubripes), zebra fish (dr, Danio rerio), amphioxus (bf, Branchiostoma floridae), sea squirt (cs, Ciona savigyni), fruit fly (dm, Drosophila melanogaster) and round worm (ce, Caenorhabditis elegans). TM represents the predicted transmembrane regions. In the sea squirt (cs, Ciona savigyni), sequence X indicates the peptide sequence X:QGQLYLLYGVLDNELYNKFVICPQTFLFG. [file 1471-2164-11-17-S4.tiff]
